# Projecting the 20-year healthcare resource burden of asthma and COPD multimorbidity: insights from Singapore for integrated chronic respiratory care in South-East Asia

**DOI:** 10.1038/s41533-026-00502-9

**Published:** 2026-04-09

**Authors:** Yah Ru Juang, Laura Huey Mien Lim, Sanjay H. Chotirmall, Kelvin Bryan Tan, Mariko Siyue Koh, John A. Abisheganaden, David B. Price, Ming-Ju Tsai, Mei Fong Liew, Pei Yee Tiew, Anthony Chau Ang Yii, Wenjia Chen

**Affiliations:** 1 Saw Swee Hock School of Public Health, National University of Singapore, Singapore, Singapore; 2 Lee Kong Chian School of Medicine, Nanyang Technological University, Singapore, Singapore; 3 Department of Respiratory and Critical Care Medicine, Tan Tock Seng Hospital, Singapore, Singapore; 4 Ministry of Health, Singapore, Singapore; 5 Department of Respiratory and Critical Care Medicine, Singapore General Hospital, Singapore, Singapore; 6 Duke-NUS Medical School, Singapore, Singapore; 7 Health Services and Outcomes Research, National Healthcare Group, Singapore, Singapore; 8 Observational and Pragmatic Research Institute, Singapore, Singapore; 9 Optimum Patient Care, Cambridge, UK; 10 Centre of Academic Primary Care, Division of Applied Health Sciences, University of Aberdeen, Aberdeen, UK; 11 Division of Pulmonary and Critical Care Medicine, Department of Internal Medicine, Kaohsiung Medical University Hospital, Kaohsiung Medical University, Kaohsiung, Taiwan; 12 Department of Internal Medicine, School of Medicine, College of Medicine, Kaohsiung Medical University, Kaohsiung, Taiwan; 13 Division of Respiratory and Critical Care Medicine, Department of Medicine, National University Hospital, National University Health System, Singapore, Singapore; 14 Department of Medicine, Yong Loo Lin School of Medicine, National University of Singapore, Singapore, Singapore; 15 Division of Respiratory and Critical Care Medicine, Integrated Medicine Programme, Alexandra Hospital, Singapore, Singapore; 16 Department of Respiratory and Critical Care Medicine, Changi General Hospital, Singapore, Singapore

**Keywords:** Diseases, Health care, Medical research, Risk factors

## Abstract

**Background:**

Asthma and chronic obstructive pulmonary disease (COPD) incur significant comorbidity and healthcare burden. However, their future economic burden remain unclear.

**Objective:**

To project 20-year (2024-2043) asthma and COPD multimorbidity costs in Singapore, illustrating broader Southeast Asian trends.

**Methods:**

Patients with asthma (all ages) or COPD ($$\ge$$40 years) were identified from Singapore’s health administrative data (2002–2019). Age- and sex-specific, disease-specific per-episode costs and annual healthcare utilisation rates (hospitalisation, emergency department, and outpatient) were estimated using generalised linear models and projected using change-point analysis. Population-level costs were projected using a probabilistic simulation model incorporating population forecasts. Costs were reported in 2023 Singaporean dollars (SGD$1 = US$0.76 = ₤0.60 = €0.69).

**Results:**

Asthma cases are projected to triple from 64,338 in 2019 to 192,409 by 2043 (95% confidence interval [CI]: 165,493–225,141), incurring $7.8 billion (95% CI: 4.4–17.1) from 2024–2043. Apart from asthma (16.4%), costs are driven by metabolic (20.0%), circulatory (14.3%), and other respiratory (9.2%) diseases, with children bearing the highest burden (girls: 39.9%; boys: 22.6%). COPD cases would grow from 8,988 in 2019 to 11,038 (95% CI: 8395–13,326) in 2043, incurring $2.4 billion (95% CI: 1.6–4.5) from 2024–2043. Apart from COPD (20.3%), metabolic (17.4%), circulatory (17.0%), and other respiratory diseases (9.8%) are the largest cost components, with elderly and adult males bearing the highest burdens (47.8% and 40.1%). In both cohorts, 20-year projected costs are dominated by outpatient (55%) and hospitalisation costs (30–40%).

**Conclusion:**

The 20-year multimorbidity costs of asthma and COPD are significant, especially in cardiometabolic comorbidities, underscoring the need for holistic, value-based care.

## Introduction

Chronic respiratory diseases (CRDs) are among the most common non-communicable diseases, with 454.6 million prevalent cases worldwide as of 2019 and ranking third among the leading causes of death^1^. Asthma and chronic obstructive pulmonary disease (COPD) respectively affect 262.4 million and 212.3 million people in 2019, representing the two most prevalent CRDs globally^1^. While asthma and COPD are both chronic inflammatory airway conditions with overlapping symptoms such as cough and shortness of breath, they differ fundamentally in disease progression and lung function trajectory^2^. Specifically, COPD is characterised by persistent and largely irreversible airflow limitation, with a progressive decline in lung function^3^. In contrast, asthma is typically associated with episodic and largely reversible airflow obstruction that generally improves with appropriate treatment^4^. Despite the considerable decline in age-standardized prevalence over the years^5^, the absolute burdens of asthma and COPD are rising, with 100 million new asthma cases expected in the next decade and 112 million new COPD cases projected between 2020 and 2050^6^. In Asia, childhood and adult asthma prevalence respectively range between 7%-13%^7^ and 0.7%-11%^8^, whereas the prevalence of COPD is estimated at 13.4% in Korea and 4.4-16.7% in China, compared with 6.5% in the U.S.^9^. Region-specific risk factors, including smoking, exposure to biomass fuel, worsening air pollution, and high body mass index (BMI) further compound the burden^10,11^.

CRDs are part of a broader multimorbidity network, often clustering with cardiorespiratory, metabolic, and neuropsychiatric disorders, driven by shared factors including ageing, systemic inflammation, environmental exposures, and use of systemic corticosteroids^12–16^. Global evidence showed that COPD is associated with a two- to five-fold elevated cardiovascular risk, including a 2.4-fold increased risk of ischemic heart disease (IHD)^17^. Findings from Canada highlighted that COPD comorbidity costs–mainly from circulatory, respiratory, and digestive disorders–double the cost of COPD itself^18^. The substantial burden of multiple chronic conditions inevitably worsen health outcomes and drive healthcare costs^18^. Notably, distinct multimorbidity clusters, such as ex-tuberculosis and diabetes-associated phenotypes, have also been identified in Asia^19^. Specifically, a low BMI was found to be associated with poorer health outcomes in COPD patients^20^, while in ethnic Chinese populations, asthma has been linked to obesity^21^ and a 1.31-fold higher risk of type 2 diabetes^22^. In Singapore, the high burden of multimorbidity in asthma and COPD patients were attributable to circulatory diseases, metabolic diseases and other respiratory conditions^23^^,^^24^. Given the treatable pulmonary and extrapulmonary traits in CRDs^25^, individualised, phenotype-based management strategies may potentially mitigate disease progression and reduce costly exacerbations.

To effectively inform evidence-based strategies and address unmet healthcare needs in CRD patients, it is crucial that the economic burden of conditions such as asthma and COPD are accurately projected. In Asia, however, up-to-date and comprehensive economic burden projections that account for the burden of comorbidities are lacking. The most recent COPD cost study in Singapore was conducted over a decade ago^26^ while the latest asthma economic analysis was limited to 500 individuals and focused solely on asthma-specific costs^27^. Furthermore, most economic burden studies in Asia are based on cross-sectional data^28,29^, surveys^30^, or simulation models^31^, many of which rely on model assumptions to capture the potentially intricate relationships between predictors. Additionally, COPD is often underdiagnosed in Asia and frequently misdiagnosed as asthma due to overlapping symptoms^32^. Given the potential diagnostic overlap, jointly evaluating the asthma and COPD populations could offer a more holistic and unbiased approach to burden estimation.

In this context, the 2022 release of Singapore national health administrative data, provided through the Trusted Research and Real World-Data Utilisation (TRUST) platform, presents a unique opportunity to address these gaps and accurately assess the burden of CRDs in a multi-ethnic Asian setting. This linked population database features an exhaustive coverage of healthcare billing claims (hospitalisation, emergency visit, primary and specialist care visit with medication dispensations) of over 4.1 million Singaporean residents across 20 years (1998–2020).

In this study, we aimed to apply data-driven forecasting and time-series analysis to project the 20-year healthcare costs of multimorbidity in patients with asthma and COPD in Singapore (2024–2043). Findings from this study would provide critical insights for the ageing Asian CRD populations with distinct clinical and comorbidity profiles.

## Methods

### Data source

To analyse past trends and forecast future burdens, we retrieved access to anonymised, individual-level linked health administrative databases of Singapore via the TRUST platform. The database included sociodemographic and claims records of over 4 million residents in Singapore with low missingness (<10%), encompassing all public primary healthcare, as well as >90% of public and private hospitals in the city-state^33^. Established for administrative and billing purposes across public healthcare institutions, the TRUST database reflected subsidised charges in public healthcare, providing accurate estimates of total (subsidised and out-of-pocket) costs. The dataset also included episode-level data on hospitalisations (1998–2020), emergency department (ED) visits (2006–2020), and public primary care outpatient visits (2012–2020), based on the respective period of available data in TRUST. All inferences, opinions, and conclusions expressed are those of the author(s) are not necessarily those of the Government, TRUST Platform developed by the Ministry of Health and Smart Nation and Digital Government Office and Synapxe, investigators or institutional partners.

### Study design and sample

We created two retrospective cohorts respectively for asthma and COPD patients (study design presented in Supplementary file Figure S1). We created an asthma cohort of all ages and a COPD cohort aged 40 years or above, using validated case definitions (i.e., having at least one inpatient visit or at least two outpatient visits on different dates, where asthma and COPD were the final diagnosis respectively, during any 12-month period (asthma case definition: sensitivity=0.632, specificity=0.997^34^, COPD case definition: sensitivity=0.850, specificity=0.784^35^). Asthma- and COPD-specific diagnoses were determined based on the International Classification of Diseases (ICD) codes (for asthma, ICD-9: 493.x except 493.2; ICD10: J45.x, J46.x; for COPD, ICD-9: 491.xx, 492.xx, 496.xx; ICD-10: J43.xx, J44.xx) recorded for each individual healthcare episode or visit. We focused on the primary diagnosis for each encounter, defined as the “most responsible diagnosis” field in hospital or ED billing claims, or the primary (and only) diagnosis field in polyclinic claims, representing the main reason for the visit. Respectively, the index date was defined as the date of the first recorded asthma- or COPD-related healthcare visit. To minimise the influence of asthma-COPD misdiagnosis, we excluded asthma patients with a diagnosis of COPD within two years of the index date, or vice versa. This “clean diagnosis window” prevents enrolling individuals who may be misdiagnosed with asthma but actually have underlying COPD, or vice versa. We analysed data according to the full available data period in the respective datasets retrieved. All subjects were followed from their index date to their date of death, loss to follow-up, or December 31, 2019, whichever came first.

### Study outcomes

The primary outcome of interest was direct medical costs, summed from hospitalisation, ED visit, and outpatient services which included medication costs. These data were directly extracted from episode-level total billing records, encompassing both subsidy and out-of-pocket expenses, and retrieved separately by healthcare setting.

In both asthma and COPD cohorts, all-cause healthcare costs were categorised into costs of the index disease (asthma or COPD) or comorbidities derived by attributing healthcare episodes to a final diagnosis of asthma/COPD, circulatory, other respiratory, digestive, infectious, nervous (including psychiatric disorders), metabolic, neoplasms, genitourinary, musculoskeletal, and other comorbidities (Supplementary file Table S1). Healthcare records with a missing diagnosis or cost value were excluded. All costs were converted to 2023 Singapore dollars (SGD$1 = US$0.76 = ₤0.60 = €0.69) based on healthcare-specific consumer price indices (CPI).

### Statistical analysis

We first presented a descriptive summary comparing the historical prevalence of asthma, COPD, and their top comorbidities – diabetes mellitus and ischemic heart disease. Using the Global Burden of Disease (GBD) estimates^36^, we reported 30-year annual trends (1990-2021) of mean prevalence in Singapore, Southeast Asia, high-income Asia Pacific, the Organisation for Economic Cooperation and Development (OECD) countries, and globally.

All statistical analyses were performed using R version 4.1.1. The unit of analysis was per-episode cost, and all analyses were performed separately by cohort (asthma, COPD), subgroup (for asthma, males or females aged 0–14, 15–64, ≥65; for COPD: males or females aged 40–64, ≥65), cost component (hospitalisation, ED visit, and outpatient visit including medication costs), and attributable disease category. Missing data (<10%) in baseline sociodemographic variables (i.e., age, sex, ethnicity, socioeconomic status (SES) and residency status) were imputed using a non-parametric random forest-based imputation algorithm.

First, age and sex-specific recent trends of healthcare utilisation rates and per-episode costs were estimated and extrapolated over the projection period i.e., 2024-2043. To model dynamic time trends, we performed change-point analysis on the historical trends of annual rate and average per-episode cost, stratified by age and sex subgroups (asthma, males or females aged 0–14, 15–64, ≥65; COPD: males or females aged 40–64, ≥65). A change-point model is a hypothesis-free statistical method for separating time trend data into a series of connected linear segments with different slopes, such that the rate of change was constant within each time-trend segment. Based on this, projections were informed by the latest stable time-trend segment^37^, which was then extended forward in linear terms over the projection horizon, assuming chronic airway diseases would incur stable healthcare utilisation under proper management. Specifically, per-episode costs across age-sex subgroups were estimated using generalised linear models (GLMs) with the normal distribution and identity link, superimposed with change-point modelling, which included the following covariates: age-sex subgroup, ethnicity, SES (measured by a modified Singapore Housing Index)^38^, residency status, calendar year, and follow-up year. Generalising estimating equation (GEE) was used to account for clustering of repeated measurements. Repeated bootstrapping was performed to obtain the mean and 95% confidence intervals (CIs) for the projected annual rates and per-episode costs. Of note, although we did not separately model end-of-life costs, the use of a marginal model allowed us to estimate population-average costs, such that high end-of-life costs among deceased patients were averaged with the costs of survivors, reflecting the overall higher cost burden and trends at the population level, particularly in COPD patients.

Based on the bootstrapped distributions, we parameterised a generalised gamma distribution for each projected rate and cost parameter across age-sex subgroups. Random sampling was then conducted in a Monte Carlo simulation with a quantile approach. Specifically, we performed random draws from rate and cost distributions that corresponded to a single quantile across all age-sex subgroups. This approach preserved the correlation in rate and cost projections across subgroups, avoiding implausible scenarios of stable trends in one group and accelerated growth in another. Lastly, we integrated population forecasts from the World Bank, which comprised of age and sex-stratified projections of population size over the 20-year projection period, to derive projected total costs at the population level.

### Ethics approval and informed consent

This study was performed in accordance with the Declaration of Helsinki. This study was approved by the National University of Singapore Institutional Review Board (NUS-IRB-2021-967). Participant consent was not required as the research involved only anonymised health administrative data.

## Results

We analysed 68,216 asthma and 18,866 COPD patients (see patient selection flowchart in Supplementary file Figure S2, patient baseline characteristics in Supplementary file Table S2). At baseline, asthma patients had a mean age of 31.8 years (standard deviation [SD] = 21.9). 48.2% were males, and ethnic composition was 50.4% Chinese, 26.8% Malays, 13.8% Indians, and 9.0% other. COPD patients had a mean age of 68.7 years (SD = 11.2), 82.9% were males, with 80.4% Chinese, 10.2% Malay, 5.4% Indian, and 4.0% other.

### Projected total cases and asthma-/COPD-specific hospitalisation rates

**Fig. 1:**
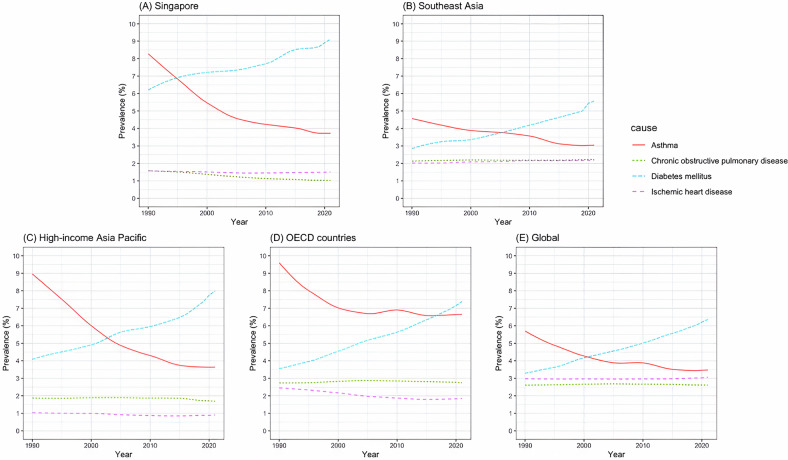
Annual prevalence of asthma, COPD, diabetes mellitus, and ischemic heart disease. Figure 1 shows the annual prevalence of asthma, chronic obstructive pulmonary disease (COPD), diabetes mellitus, and ischemic heart disease across five regions: (**A**) Singapore, (**B**) Southeast Asia, (**C**) high-income Asia Pacific, (**D**) OECD countries, and (**E**) global estimates.

Based on GBD estimates, from 1990 to 2021, asthma prevalence declined across all regions, with the largest declines observed in Singapore (from 8.3% to 3.7%) and high-income Asia Pacific countries (from 9.0% to 3.6%). More moderate declines were seen in Southeast Asia (4.6% to 3.0%), OECD countries (9.6% to 6.7%), and globally (5.7% to 3.5%). In contrast, diabetes prevalence rose steadily during the same period. COPD and ischaemic heart disease prevalence remained relatively stable from 1990 to 2021, ranging from 1.0% to 2.7% and 1.5% to 3.1%, respectively, across regions [Fig. 1].

**Fig. 2:**
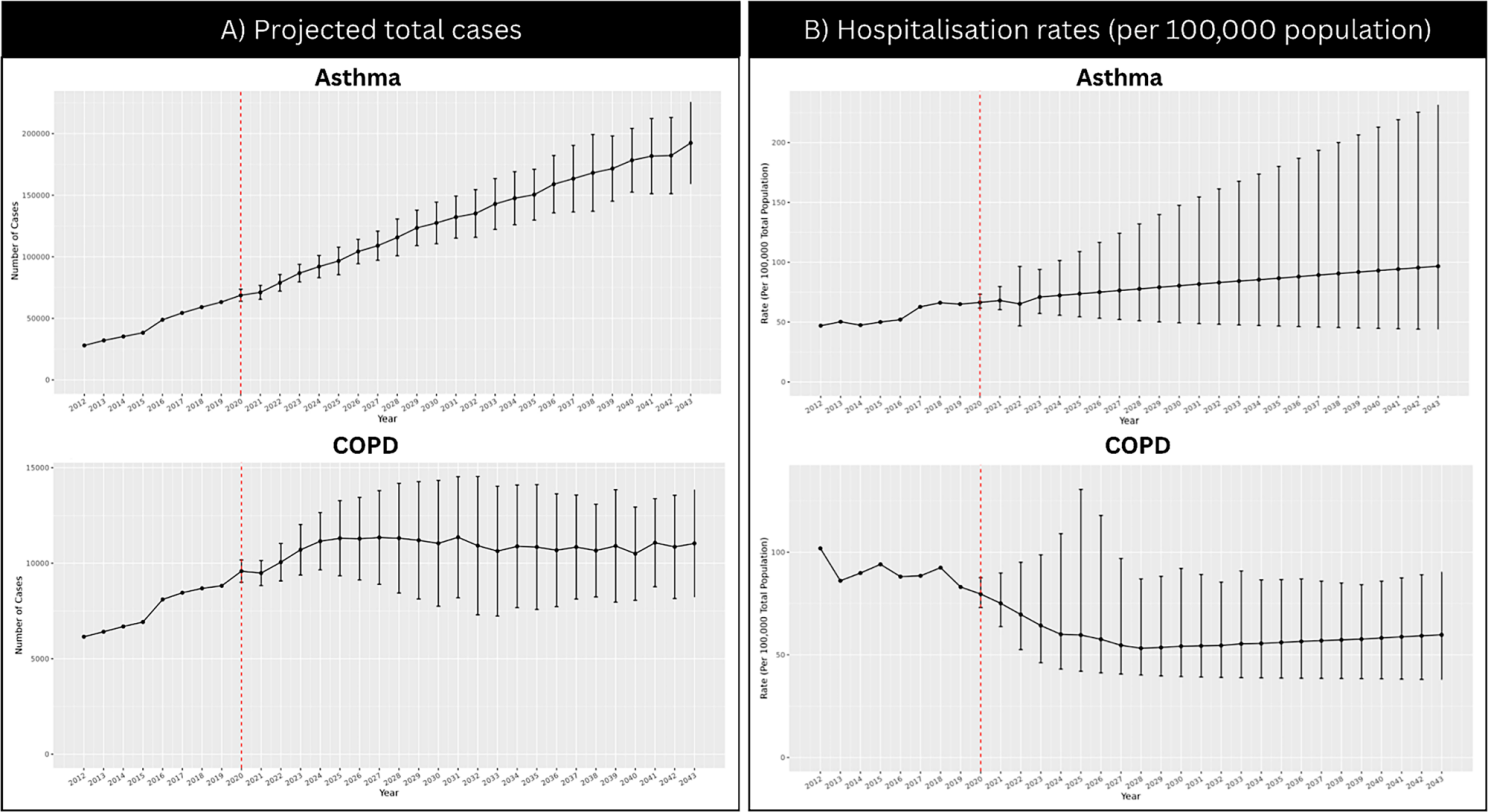
Projected total cases and asthma/COPD-specific hospitalisation rates (per 100,000 total population). Figure 2 shows the projected number of asthma and COPD cases (**A**) and disease-specific hospitalisation rates (per 100,000 total population) (**B**) from 2024 to 2043. The vertical dashed line in red represents the start of projection (i.e., year 2020). The error bars represent 95% confidence intervals (Cis) around the projection estimates.

Figure 2 presents projected asthma and COPD cases (panel A) and hospitalisation rates (panel B) in Singapore’s hospitals (including specialist clinics) and primary care. Between 2019 and 2024, asthma cases increased from 64,338 to 92,039, while COPD cases only marginally grew from 8,988 to 10,152. By 2043, asthma cases are projected to reach 192,409 (95% CI: 165,493-225,141; 109.1% increase) while COPD cases will remain stable at 11,038 (95% CI: 8,395-13,326; 8.7% increase). Over the next 20 years till 2043, most asthma cases will occur in adult females (32.7%) and males (25.2%), while COPD will predominately affect middle-aged males (88.8%) (Supplementary file Table S3).

As of 2024, the average asthma-specific hospitalisation rate is projected to reach 72.3 per 100,000 (95% CI: 55.7-101.4), highest among paediatric males (192.9 per 100,000) and females (130.0 per 100,000) (Supplementary file Table S4). By 2043, this rate is expected to rise by 34% to 96.7 per 100,000 (95% CI: 43.8-231.6), mainly driven by children, reaching 321.6 and 243.2 per 100,000, in boys and girls, respectively.

As of 2024, the average COPD-specific hospitalisation rate is projected at 60.0 per 100,000 (95% CI: 43.1-109.1), highest among middle-aged (124.5 per 100,000) and elderly males (94.4 per 100,000). By 2043, the overall rate is expected to remain stable at 59.8 per 100,000 (95% CI: 37.9-90.5), with middle-aged males reaching 200.5 per 100,000 (Supplementary file Table S4).

### Projected 20-year costs in asthma and COPD patients, by component and multimorbidity

**Fig. 3:**
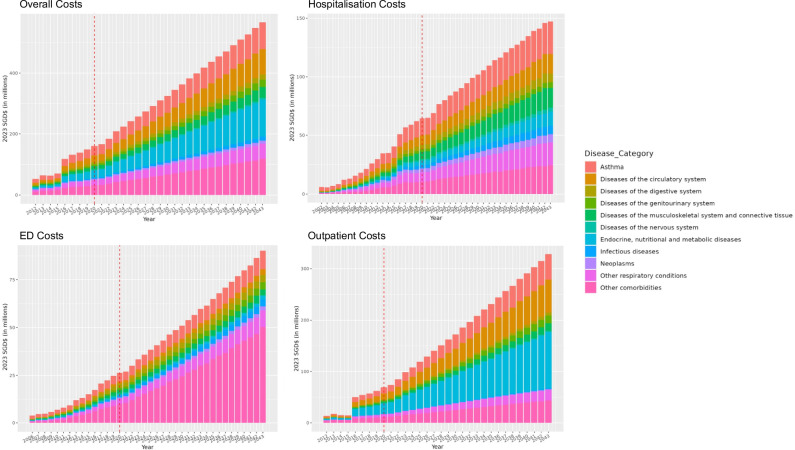
Projected costs of multimorbidity in asthma patients by cost components. Figure 3 shows the projected 20-year costs in asthma patients from 2024 to 2043, split by disease category. Panels A, B, C, and D respectively show the projected total costs, hospitalisation costs, ED visit costs, and outpatient visit costs. The vertical dashed line in red represents the start of projection (i.e., year 2020). All costs were measured in 2023 Singaporean dollars (SGD$1 = US$0.76 = ₤0.60 = €0.69).

As shown in Fig. 3, as of 2024, the total direct cost in asthma patients was $223.9 (95% CI: $165.1-342.2) million, of which $80.0 (35.7%) million, $35.8 million (16.0%), and $108.2 million (48.3%) were attributable to costs of hospitalisation, ED visits, and outpatient (primary and specialist care) visits with medication dispensations, respectively.

In asthma patients, the projected 20-year total cost was $7.8 (95% CI: $4.4-17.1) billion, mainly due to outpatient (55.0%, $4.3 [95% CI: $2.2-10.5] billion) and hospitalisation costs (29.3%, $2.3 [95% CI: $1.3-4.7] billion). The projected increase in aggregate costs is mainly driven by increasing rates of hospitalisation and outpatient visits rather than inflation of per-episode costs, as shown in Supplementary file Figures S3 and S5. The most costly conditions were metabolic diseases (20.0%, $1.6 [95% CI: $0.5-5.2] billion), asthma (16.4%, $1.3 [95% CI: $0.9-2.1] billion), circulatory diseases (14.3%, $1.1 [95% CI: 0.6-2.7] billion), and non-asthma respiratory conditions (9.2%, $0.7 [95% CI: $0.4-1.3] billion) [Fig. 3].

**Fig. 4:**
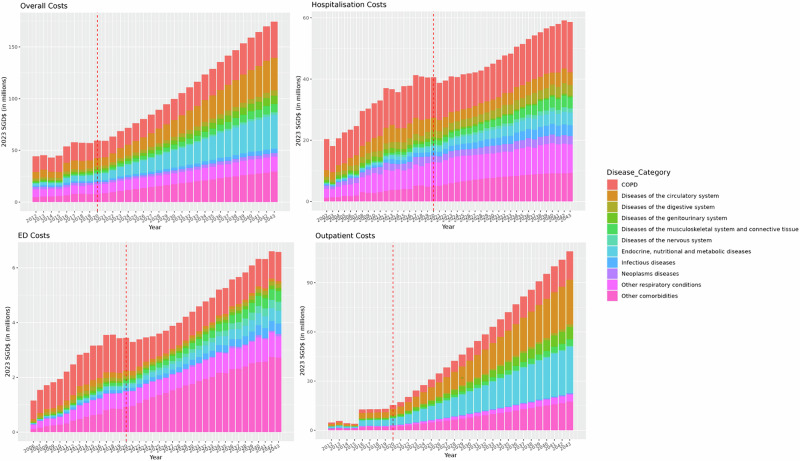
Projected costs of multimorbidity in COPD patients by cost components. Figure 4 shows the projected 20-year costs in COPD patients from 2024 to 2043, split by disease category. Panels A, B, C, and D respectively show the projected total costs, hospitalisation costs, ED visit costs, and outpatient visit costs. The vertical dashed line in red represents the start of projection (i.e., year 2020). All costs were measured in 2023 Singaporean dollars (SGD$1 = US$0.76 = ₤0.60 = €0.69).

In Fig. 4, as of 2024, the total cost in COPD patients was $71.7 (95% CI: $55.3-103.1) million. Of these, $40.7 million (56.8%), $3.5 million (4.9%), and $27.5 million (38.3%) were attributable to costs of hospitalisation, ED visits, and outpatient visits with medication dispensations, respectively.

In COPD patients, the projected 20-year total cost was $2.4 (95% CI: $1.6-4.5) billion, mainly driven by outpatient visit costs (54.8%, $1.3 [95% CI: $1.0-1.8] billion) and hospitalisation costs (41.0%, $1.0 [95% CI: $0.5-2.5] billion). The projected increase in aggregate costs are mainly driven by increasing rates outpatient visits among elderly males, rather than inflations of per-episode costs, as shown in Supplementary file Figures S4 and S6. The most costly conditions were COPD (20.3%, $0.5 [95% CI: $0.3-0.8] billion), metabolic diseases (17.4%, $0.4 [95% CI: $0.3-0.6] billion), circulatory diseases (17.0%, $0.4 [95% CI: $0.4-0.5] billion), and non-COPD respiratory diseases (9.8%, $0.2 [95% CI: $0.1-0.8] billion) [Fig. 4].

### Projected 20-year costs in asthma and COPD patients, by age and sex

**Fig. 5:**
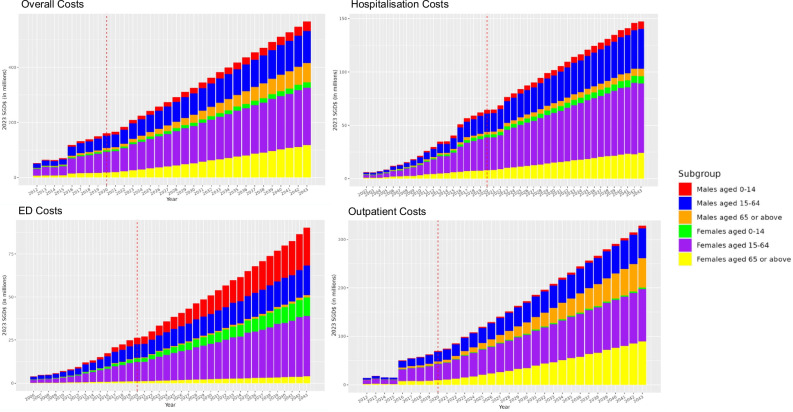
Projected total costs in asthma patients by age-sex subgroups and cost components. Figure 5 shows the projected 20-year total costs in asthma patients from 2024 to 2043, split by age-sex group and cost components (hospitalisations, ED, and outpatient visits). The vertical dashed line in red represents the start of projection (i.e., year 2020). All costs were measured in 2023 Singaporean dollars (SGD$1 = US$0.76 = ₤0.60 = €0.69).

Of the projected 20-year total cost in asthma patients, most of cost burden will be incurred by adult females (39.9%, $3.1 [95% CI: $2.1-5.1] billion) and adult males (22.6%, $1.8 [95% CI: $1.2-2.9] billion). For projected hospitalisation costs, adult females (45.4%, $1.0 billion) and males (25.5%, $0.6 billion) contributed the most. For ED costs, adult females (40.0%, $0.5 billion) and paediatric males (22.4%, $0.3 billion) contributed the most. For outpatient with medication costs, adult females (36.8%, $1.6 billion) and elderly females (23.3%, $1.0 billion) contributed the most [Fig. 5].

Particularly in the paediatric asthma group, the projected 20-year costs were estimated at $0.7 billion (boys: $0.5 billion; girls: $0.3 billion), mainly driven by ED costs (boys: 37.0%, $0.3 billion; girls: 18.0%, $0.1 billion) and hospitalisation costs (boys: 15.1%, $0.1 billion; girls: 13.6%, $0.1 billion).

**Fig. 6:**
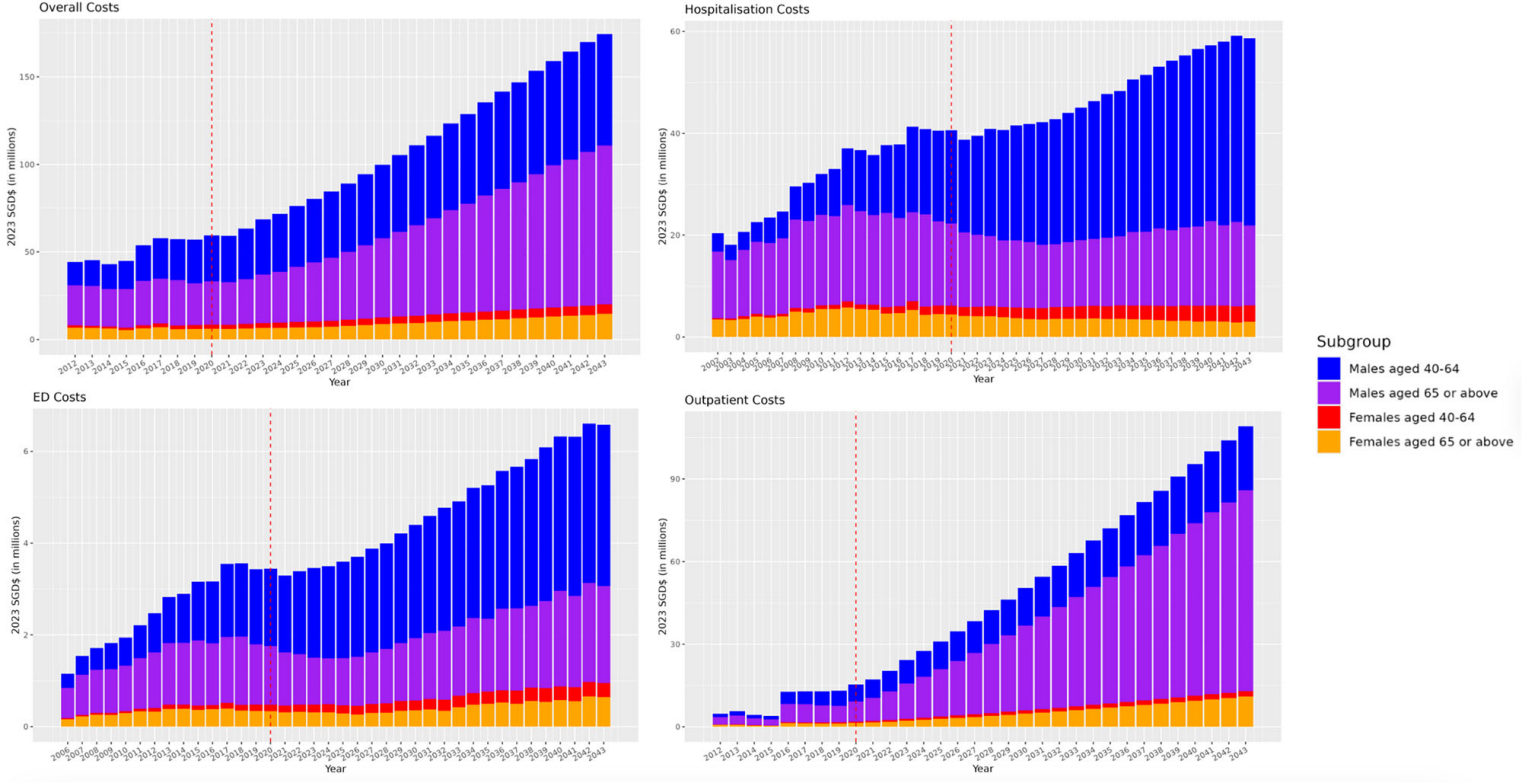
Projected total costs in COPD patients by age-sex subgroups and cost components. Figure 6 shows the projected 20-year total costs in COPD patients from 2024 to 2043, split by age-sex group and cost components (hospitalisations, ED, and outpatient visits). The vertical dashed line in red represents the start of projection (i.e., year 2020). All costs were measured in 2023 Singaporean dollars (SGD$1 = US$0.76 = ₤0.60 = €0.69).

In COPD patients, most of the projected 20-year total costs will be incurred by elderly males (47.8%, $1.2 [95% CI: $0.7-2.5] billion) and middle-aged males (40.1%, $1.0 [95% CI: $0.7-1.4] billion). For projected hospitalisation costs, middle-aged males (59.3%, $0.6 billion) and elderly males (25.5%, $0.3 billion) accounted for the most. For ED costs, middle-aged males (55.4%, $56.0 million) and elderly males (30.7%, $31.0 million) contributed the most. For outpatient with medication costs, elderly males (63.5%, $0.8 billion) and middle-aged males (24.7%, $0.3 billion) contributed the most [Fig. 6].

## Discussion

To our knowledge, this is the first data-driven forecasting study in a non-Western setting to project the multimorbidity cost burden of asthma and COPD patients using comprehensive national health administrative data from Singapore, a multi-ethnic Asian country. In Singapore’s hospital and primary care settings, over the next 20 years, while COPD cases appeared to remain stable, asthma cases are projected to double, with the greatest increase in children. From 2024 to 2043, asthma-related hospitalisation rates are projected to increase by one-third, while COPD rates will remain largely change, and the projected total cost in asthma and COPD patients are, respectively, $7.8 billion and $2.4 billion. Of note, the projected burdens of asthma may be underestimated due to substantial underdiagnosis and the absence of private primary care data from our analysis. In Singapore, 55% of chronic care are provided by private general practitioners (GPs)^39^. In particular, the disproportionately high hospitalisation costs among adult females with asthma and the underrepresentation of women (<10%) in COPD patients over the next 20 years merits further investigations.

The projected rise in asthma prevalence may be explained by population dynamics and an increasing burden of allergic airway diseases in Southeast Asia, partly driven by house dust mite sensitisation in tropical urban environments^40^. Singapore, in particular, exhibits high rates of house dust mite allergy^41^, especially among children, which may explain the rapid anticipated growth in paediatric asthma cases. In fact, a comparison among Asian countries showed that the highest rates of house dust mite sensitisation in allergic individuals were reported in Singapore (up to >90%), followed by Taiwan (85-90%) and South India (89.7%)^41^. More broadly, recent reports indicate that allergic diseases in the Asia-Pacific region have reached their highest level in the past few decades^42^, with similar trends observed across tropical Southeast Asia^43^. Warm and humid climates in these regions promote persistent exposure to region-specific aeroallergens, contributing to increased asthma prevalence and symptom burden^44^. Unlike temperate regions, allergen profiles in tropical and subtropical areas, including parts of China, are dominated by mould spores that thrive under high humidity and temperature^45^, contributing to acute childhood asthma exacerbations and short-term increases in asthma-related hospital admissions, thereby complicating disease management in these populations^45,46^.

Additionally, a considerable fraction of adult females with COPD may have been misclassified as asthma patients^47^, leading to suboptimal COPD treatment and unnecessary asthma-related healthcare utilisation, as the high asthma hospitalisation costs in adult female asthmatics had coincided with the marked under-representation of women in COPD diagnoses (<10% projected over the next 20 years). In our cohort, COPD patients had a markedly older age at diagnosis (mean 68.7 years), a strikingly low overall prevalence (<0.5%), and a highly imbalanced male-to-female ratio (9:1), which contrasts with global, mainly Western-based evidence (mean age, 57.3^48^, prevalence, ~10.6%^6^, and a sex ratio 1:1 in global estimates^49^), despite similar trends in population ageing amid declining smoking rates. Although smoking rates in Singapore have declined by 20% to 10.6% in 2019^50^, which may partly explain the country’s low COPD prevalence, the consistently higher number of diagnosed asthma cases relative to COPD in our study more likely reflects asthma overdiagnosis, substantial underdiagnosis or delayed diagnosis of COPD, and the exclusion of asthma-COPD overlap cases. This imbalance may stem from diagnostic biases, particularly the overreliance on smoking history for COPD identification^51^, which may inadvertently exclude non-smokers, particularly females. Other contributing factors likely include limited pulmonary function testing in primary care^52^ and diagnostic stigma^53^, both of which can result in delayed diagnosis and reduced mid-term survival^54^. Meanwhile, both asthma and COPD are systemic conditions that, when undertreated or mismanaged, such as with prolonged oral corticosteroid (OCS) use in asthma patients, can accelerate comorbidity development, increasing hospitalisations and healthcare costs^55^. Also, most projected COPD cases occur in males aged 40–64, likely reflecting early disease influenced by genetic predisposition (e.g., alpha-1 antitrypsin deficiency^56^) and early-life exposures to air pollution or occupational hazards^57^. These findings highlight the urgent need for timely diagnosis, broader access to confirmatory testing in primary care, and more personalised, evidence-based treatment strategies to mitigate disease progression and reduce downstream health and economic burdens^58^.

Top drivers of future multimorbidity costs in this study likely reflect genetic dispositions, population-specific influences in Asia, and suboptimal CRD management. Projected metabolic disease costs are expected to match COPD costs in COPD patients, and exceed asthma costs by 1.2 times in asthmatics, primarily driven by outpatient services, with cardiovascular conditions similarly burdensome. This is partly influenced by ethnic traits such as increased visceral adiposity may heighten cardiometabolic comorbidities risk. In fact, cardiometabolic diseases dominate chronic disease prevalence in Singapore, with chronic kidney disease, hypertension, and lipid disorders being the most prevalent individually and as multimorbidity combinations^59,60^. Moreover, these risks are compounded by Asian-specific disease phenotypes and metabolic susceptibilities, including a higher propensity for visceral adiposity and insulin resistance^61^, which contribute to high burden of diabetes and metabolic multimorbidity observed among asthma patients in Singapore, as well as obesity in non-Chinese female asthmatics^21^, which is associated with poorer asthma control. In COPD, a history of tuberculosis in COPD further exacerbates metabolic and respiratory burden^19^. In severe asthma, OCS overuse, a growing concern in Singapore^55^, may trigger or exacerbate OCS-related comorbidities like type 2 diabetes, obesity, gastroesophageal reflux disease (GERD), and psychiatric disorders. Similarly, steroid overuse in COPD also contributes to comorbidities, including cardiovascular and cerebrovascular disease^15^, driving higher healthcare costs in asthma and COPD patients^62,63^. Addressing these surging multimorbidity burdens require integrated care, including nutritional interventions, adjusted steroid use in severe asthma and COPD treatment, and routine metabolic monitoring in COPD management.

To address the unsustainable trajectory of CRD, Singapore must shift from a reactive to a preventive care model, in alignment with the national Healthier SG strategy^64^, which aims to keep people healthier by focusing on prevention and coordinated care through community- and primary care-led initiatives. Efforts should focus on early diagnosis and management of asthma and COPD in primary care using spirometry, value-based care, community screening, patient self-management plans, integrated care pathways^64^. Existing community asthma and COPD programmes^65,66^ can address care gaps, such as suboptimal medication use and missed follow-ups^67,68^. Aligned with Healthier SG, scaling preventive care through integrated, value-based models can reduce preventable exacerbations, improve long-term outcomes, and support sustainable care. In parallel, incorporating excess comorbidity-related costs in cost-effectiveness evaluations of emerging therapeutics (e.g., biologics, inhaled triple therapy) and harnessing artificial intelligence (AI)-enabled precision medicine tools to identify high-risk patients can further optimise management and reduce hospitalisations. Lastly, investigating environmental determinants, particularly air quality and climate change, is critical, as they affect respiratory disease risk, treatment response, and health outcomes. Reducing ambient pollutant exposure could potentially alleviate respiratory morbidity and achieve long-term public health gains.

Our study has several key strengths. The extensive health administrative data allowed us to perform a population-based analysis and derive granular cost estimates. The use of data-driven forecasting techniques also allowed us to leverage observed trends to capture the net effect of known and unknown risk factors in projecting future burden, reducing reliance on assumptions. Coupled with time-series analysis^69^, this method reflects real-world healthcare complexities, including evolving treatments, misdiagnosis, and delayed care contributing to high-cost acute episodes. However, several study limitations have to be acknowledged. First, the absence of private primary care data may underestimate the true burden, particularly for asthma, often managed outside the public system. Notably, outpatient cost only include specialist and public primary care, excluding patients in private GPs clinics. Second, key variables such as OCS use and smoking status were unavailable in the MOH TRUST dataset, which limited causal inference amid unmeasured confounding effects. Third, the study period (2012–2019) may be too short to capture long-term outpatient care trends, and medication costs may be underestimated if prescriptions were filled outside public care system (e.g., pharmacies). Fourth, despite a two-year washout, some asthma-COPD misclassification may persist. Fifth, asthma-COPD overlap cases may exhibit distinct patterns in multimorbidity burdens which were not explored. Sixth, the descriptive summary of asthma and COPD prevalence included 2020-2021 data, which might underestimate their prevalence due to stricter control measures during the COVID-19 pandemic^70^. Additionally, clinical biomarkers and spirometry results were not available in the health administrative data, which prevented rigorous assessment of asthma and COPD diagnoses despite the use of validated case definitions. Seventh, our models did not account for individual healthcare subsidies, which could potentially bias outcome predictions as affordability is an influencing factor of health-seeking behaviour. However, this limitation may be partially mitigated by adjusting for socioeconomic status in the models. Eighth, our projections are primarily data-driven and do not explicitly model upstream risk factors such as smoking patterns or environmental exposures; thus, they do not replace model-based projections that incorporate these determinants. Nevertheless, our approach provides complementary and unique strengths by quantifying comprehensive healthcare utilisation and multimorbidity-related costs using real-world data. Lastly, while change-point analysis captured shifts in historical disease and healthcare trends, uncertainty in Singapore’s population forecasts, due to migration, fertility, and mortality, may slightly affect our long-term projections’ accuracy. Moreover, under the data-driven forecasting approach of this study, future healthcare utilisation rates and costs were projected based on the latest stable trends from change-point analysis. However, future trends of healthcare utilisation are subject to policy influence, and correspondingly, the level of uncertainty around the projection estimates would increase over the projection time horizon, as reflected by the widening confidence intervals.

In summary, respiratory conditions account for severe hospitalisation burdens in CRD patients. Aside from asthma and COPD themselves, metabolic and circulatory diseases are leading components of total costs. A comprehensive, forward-looking approach integrating accurate diagnosis, equitable access to advanced treatment, and tailored preventive strategies is essential to mitigate the long-term CRD burden in Singapore.

## Supplementary information

Supplementary Information

## Data Availability

The dataset used in this study are analysed in a secure environment and are not available for external request.

## Code availability

The underlying code for this study is not publicly available but may be made available to qualified researchers on reasonable request from the corresponding author.

## Declarations

This study made use of data generated by the Ministry of Health and/or anonymised data retrieved from government administrative and health records. This study was supported by the Trusted Research and Real-World-Data Utilisation and Sharing Tech platform (“TRUST Platform”) jointly developed by the Ministry of Health and Smart Nation and Digital Government Office and Synapxe. The views expressed are those of the author(s) are not necessarily those of the Government, TRUST Platform developed by the Ministry of Health and Smart Nation and Digital Government Office and Synapxe, investigators or institutional partners. We thank all participants and research staff who made the study possible.
