# Supplementary material for: Projecting the 20-year healthcare resource burden of asthma and COPD multimorbidity: insights from Singapore for integrated chronic respiratory care in South-East Asia
Source: NPJ Prim Care Respir Med. 2026 Apr 9;36:36. doi: 10.1038/s41533-026-00502-9 (PMC13270152; doi:10.1038/s41533-026-00502-9)
Supplement: Supplementary file 1 — Supplementary Information [file 41533_2026_502_MOESM1_ESM.docx]

**Supplementary file**

**Projecting the 20-Year Healthcare Resource Burden of Asthma and COPD Multimorbidity: Insights from Singapore for Integrated Chronic Respiratory Care in South-East Asia**

Yah Ru Juang BSc^1¶^, Laura Huey Mien Lim MSc^1¶^, Sanjay H. Chotirmall MD PhD^2,3^ , Kelvin Bryan Tan PhD^1,4^, Mariko Siyue Koh MBBS MRCP^5,6^, John Abisheganaden MBBS MRCP MMed FAMS FRCP^2,7^, David Price FRCGP^8-10^, Ming-Ju Tsai MD PhD^11,12^, Mei Fong Liew MBBS MRCP MMed^13-15^, Pei Yee Tiew MD PhD^2,5,6^, Anthony Chau Ang Yii MB BChir MA MRCP MPH^16^, Wenjia Chen PhD^1^

^¶^ Co-first authors

^1^ Saw Swee Hock School of Public Health, National University of Singapore, Singapore, Singapore

^2^ Lee Kong Chian School of Medicine, Nanyang Technological University, Singapore, Singapore

^3^ Department of Respiratory and Critical Care Medicine, Tan Tock Seng Hospital, Singapore

^4^ Ministry of Health, Singapore, Singapore

^5^ Department of Respiratory and Critical Care Medicine, Singapore General Hospital, Singapore, Singapore

^6^ Duke-NUS Medical School, Singapore

^7^ Health Services and Outcomes Research, National Healthcare Group, Level 4 @ NSC, 1 Mandalay Rd, Singapore, 308205, Singapore

^8^ Observational and Pragmatic Research Institute, Singapore, Singapore

^9^ Optimum Patient Care, Cambridge, UK

^10^ Centre of Academic Primary Care, Division of Applied Health Sciences, University of Aberdeen, Aberdeen, UK

^11^Division of Pulmonary and Critical Care Medicine, Department of Internal Medicine, Kaohsiung Medical University Hospital, Kaohsiung Medical University, Kaohsiung, Taiwan

^12^ Department of Internal Medicine, School of Medicine, College of Medicine, Kaohsiung Medical University, Kaohsiung, Taiwan

^13^ Division of Respiratory and Critical Care Medicine, Department of Medicine, National University Hospital, National University Health System, Singapore, Singapore

^14^ Department of Medicine, Yong Loo Lin School of Medicine, National University of Singapore, Singapore, Singapore

^15^ Division of Respiratory and Critical Care Medicine, Integrated Medicine Programme, Alexandra Hospital, Singapore, Singapore

^16^ Department of Respiratory and Critical Care Medicine, Changi General Hospital, Singapore, Singapore

* Correspondence: Laura Huey Mien Lim; National University of Singapore, Saw Swee Hock School of Public Health, 12 Science Drive 2, Singapore 117549; Email: [e0105060@u.nus.edu](mailto:e0105060@u.nus.edu)

**Table S1: Major disease categories (Asthma and COPD patients)**

| **Category** | **ICD-9 Codes** | **ICD-10 Codes** |
| --- | --- | --- |
| **Circulatory Diseases** | 390–459 | I00–I99 |
| **Other Respiratory Diseases** | Asthma: 460–519 (Excl. 493)  COPD: 460–519 (Excl. 491–492, 496) | Asthma: J00–J99 (Excl. J45–J46)  COPD: J00–J99 (Excl. J43–J44) |
| **Digestive Diseases** | 520–579 | K00–K93 |
| **Infectious Diseases** | 001–139 | A00–B99 |
| **Neuropsychiatric Diseases** | 290–389 | G00–G99, F00–F99 |
| **Metabolic Diseases** | 240–279 | E00–E99 |
| **Neoplasms** | 140–239 | C00–D49 |
| **Genitourinary Diseases** | 580–629 | N00–N99 |
| **Musculoskeletal Diseases** | 710–739 | M00–M99 |
| **Other comorbidities** | All excluding circulatory, other respiratory, digestive, infectious, nervous system, endocrine and metabolic, neoplasms, genitourinary and musculoskeletal diseases | All excluding circulatory, other respiratory, digestive, infectious, nervous system, endocrine and metabolic, neoplasms, genitourinary and musculoskeletal diseases |

**Figure S1: Schematic Diagram of Study Design**


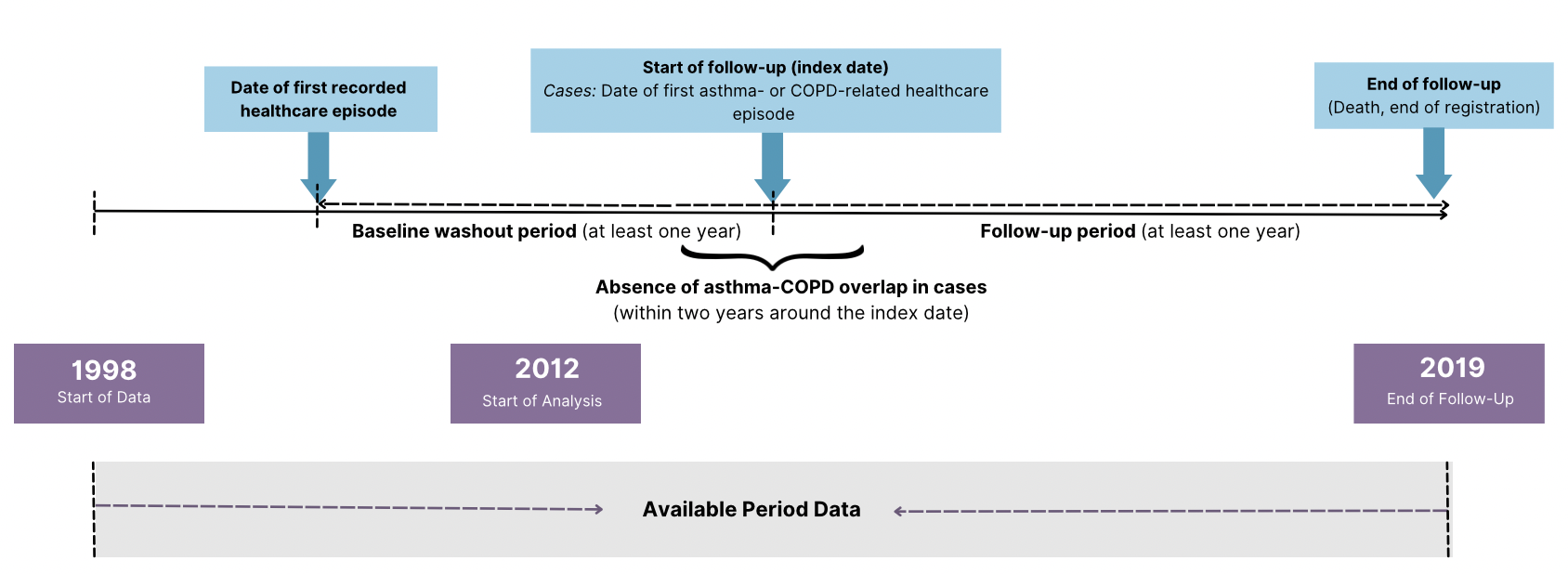


**Figure S2: Patient Flowchart**

1. Asthma Patients


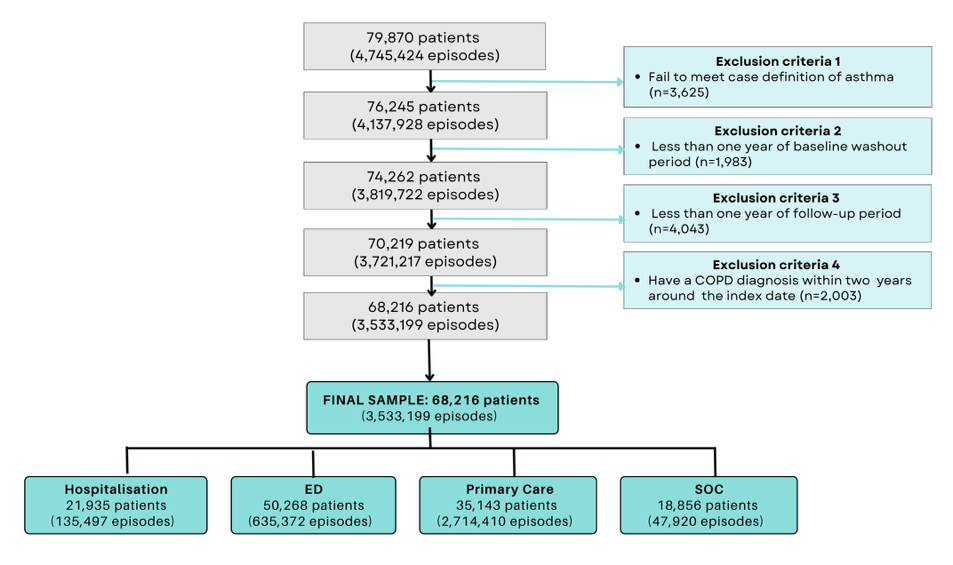


1. COPD Patients


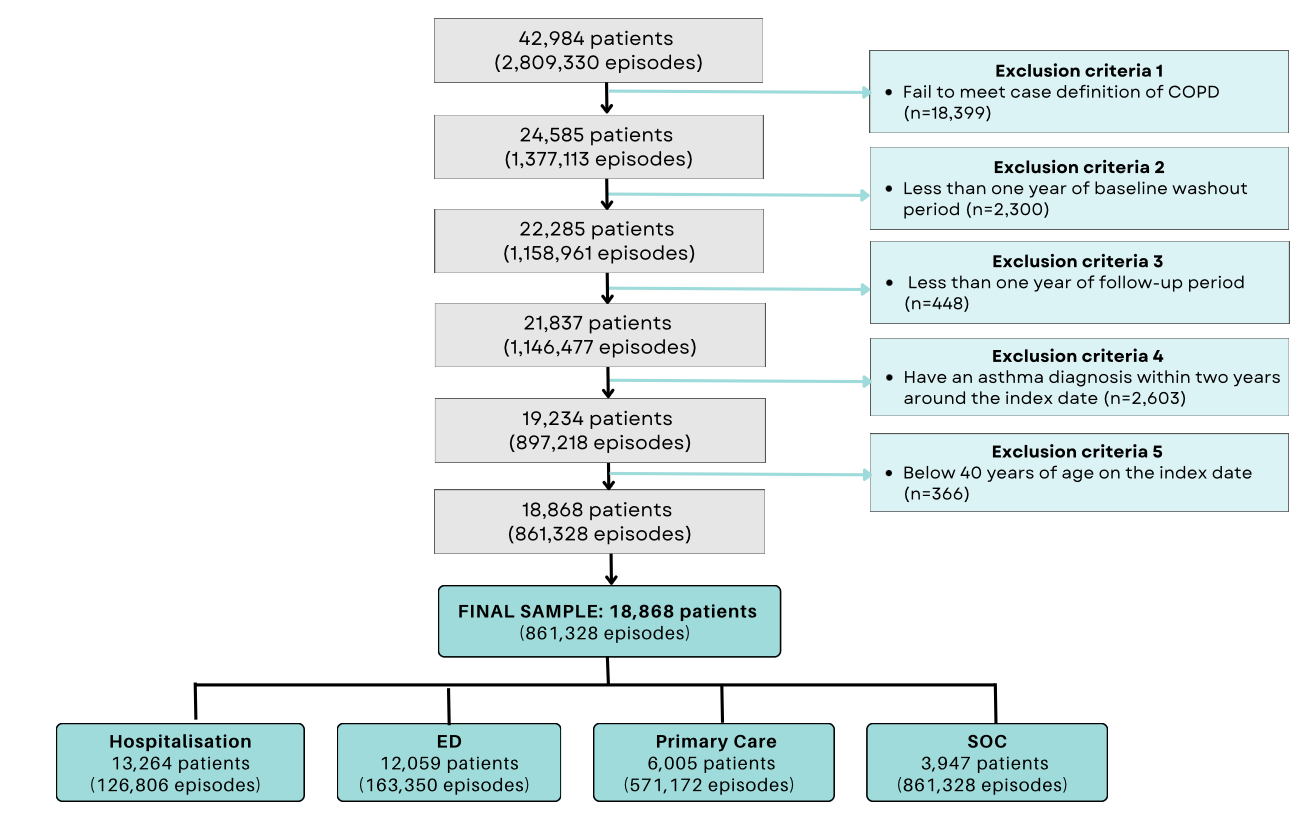


**Table S2: Baseline characteristics of patient sample**

| **CHARACTERISTICS** | **Asthma** | | | **COPD** | | |
| --- | --- | --- | --- | --- | --- | --- |
|  | **Total**  **N = 68,216** | **Pediatric (0-14 years)**  **N=16,293** | **Adult (>15 years)**  **N=51,923** | **Total**  **N = 18,886** | **Middle aged (40-64 years)**  **N=6,271** | **Elderly (>65 years)**  **N=12,595** |
| Age, mean (SD) | 31.8 (21.9) | 4.5 (4.5) | 40.4 (17.7) | 68.7 (11.2) | 56 (6.4) | 75 (6.8) |
| Age group, n (%)    0-14  15-64  40-64  65 or above | 16,293 (23.9)  46,224 (67.8)  -  5,699 (8.4) | 16,293 (100)  0 (0)  -  0 (0) | 0 (0)  46,224 (89)  -  5,699 (11) | -  -  6,271 (33.2)  12,595 (66.8) | -  -  6,271 (100)  - | -  -  -  12,595 (100) |
| Gender, n (%)  Male  Female | 32,907 (48.2)  35,309 (51.8) | 9,926 (60.9)  6,367 (39.1) | 22,981 (44.3)  28,942 (55.7) | 16,252 (82.9)  3,363 (17.1) | 5,700 (90.9)  571 (9.1) | 9,956 (79.0)  2,639 (21.0) |
| Ethnicity  Chinese  Indian  Malay  Others | 34,365 (50.4)  9,446 (13.8)  18,285 (26.8)  6,120 (9) | 8,072 (49.5)  1,762 (10.8)  5,238 (32.1)  1,221 (7.5) | 26,293 (50.6)  7,684 (14.8)  13,047 (25.1)  4,899 (9.4) | 15,159 (80.4)  1,013 (5.4)  1,993 (10.2)  761 (4.0) | 4,574 (72.9)  510 (8.1)  872 (13.9)  315 (5.0) | 10,585 (84.0)  503 (4.0)  1,061 (8.4)  446 (3.5) |
| SES, n (%)  Low  Middle  High | 22,461 (32.9)  36,977 (54.2)  8,778 (12.9) | 5,434 (33.4)  8,854 (54.3)  2,005 (12.3) | 17,027 (32.8)  28,123 (54.2)  6,773 (13) | 7,596 (40.3)  9,392 (49.8)  1,878 (10.0) | 2,545 (40.6)  3,066 (48.9)  660 (10.5) | 5,051 (40.1)  6,326 (50.2)  1,218 (9.7) |
| Residency Status, n (%)  Resident  Non-resident | 64,003 (93.8)  4,213 (6.2) | 15,196 (93.3)  1,097 (6.7) | 44,807 (94)  3,116 (6) | 18,011 (95.5)  855 (4.5) | 6,048 (96.4)  223 (3.6) | 11,963 (95.0)  632 (5.0) |
| Baseline comorbidities, n (%)  Circulatory  Digestive  Genitourinary  Infectious  Musculoskeletal  Neoplasms  Nervous  Metabolic  Non-asthma/Non-COPD respiratory  Other comorbidities | 14,506 (21.1)  18,264 (26.8)  13,286 (19.5)  16,818 (24.7)  19,120 (28.0)  2,891 (4.2)  9,321 (13.7)  16,235 (23.8)  29,564 (43.3)  30,785 (45.1) | 325 (2)  3,160 (19.4)  1,352 (8.3)  4,306 (26.4)  1,750 (10.7)  84 (0.5)  1,339 (8.2)  416 (2.6)  5,915 (36.3)  5,565 (34.2) | 14,080 (27.1)  15,104 (29.1)  11,934 (23)  12,512 (24.1)  17,370 (33.5)  2,807 (5.4)  7,982 (15.4)  15,819 (30.5)  23,649 (45.5)  25,220 (47.6) | 5,073 (26.9)  3,663 (19.4)  2,955 (15.7)  2,209 (11.7)  3,356 (17.8)  760 (4.0)  1,192 (6.3)  4,188 (22.2)  5,737 (30.4)  6,070 (32.2) | 1,972 (31.4)  1,558 (24.8)  1,039 (16.6)  1,024 (16.3)  1,487 (23.7)  267 (4.3)  528 (8.4)  1,704 (27.2)  2,402 (38.3)  2,495 (39.8) | 3,101 (24.6)  2,105 (16.7)  1,916 (15.2)  1,185 (9.4)  1,869 (14.8)  493 (3.9)  664 (5.3)  2,484 (19.7)  3,335 (26.5)  3,575 (28.4) |
| Baseline HCU per PY, mean (SD)  Hospitalisation  ED visit  Outpatient visit | 0.9 (3.9)  3.1 (9.3)  19.1 (37.3) | 0.6 (2.5)  2.9 (8)  6.2 (17.4) | 1 (4.2)  3.1 (9.7)  23.1 (40.8) | 2.1 (6.4)  2.9 (9.2)  13.7 (34.4) | 2.7 (7.5)  4.1 (12.3)  17.6 (38.5) | 1.9 (5.8)  2.3 (7.1)  11.7 (32.0) |
| Baseline hospital bed-days per PY, mean (SD) | 4 (21.7) | 2 (11.5) | 4.7 (24) | 12.8 (40.9) | 14.8 (45.1) | 11.9 (38.6) |

**Abbreviations:** ED: emergency department; HCU: healthcare utilisaiton; SES: socioeconomic status; SD: standard deviation

**Note**: All costs were measured in 2023 Singaporean dollars (SGD$1=US$0.76=₤0.60=€0.69).

**Table S3: Projected asthma and COPD cases**

| **Asthma** | | |
| --- | --- | --- |
| Number of cases (95% CI) | **2024** | **2043** |
|  | 92,039 (84,359-99,863) | 192,409 (165,493-225,141) |
| **Age-sex subgroups** | | |
| Males aged 0-14 | 13,889 (12,977-14,835) | 28,232 (24,766-31,016) |
| Females aged 0-14 | 8,716 (6,678-10,655) | 17,737 (12,323-22,194) |
| Males aged 15-64 | 27,094 (18,377-34,853) | 48,442 (28,796-68,302) |
| Females aged 15-64 | 33,954 (32,2) | 62,919 (55,764-67,796) |
| Males aged 65 or above | 2,854 (387-4,714) | 11,735 (887-26,217) |
| Females aged 65 or above | 5,531 (2,249-8.726) | 23,343 (5,312-43,456) |
| **COPD** | | |
| Total number of cases (95% CI) | **2024** | **2043** |
|  | 10,152 (8,864-12,619) | 11,038 (8,395-13,326) |
| **Age-sex subgroups** | | |
| Males aged 40-64 | 5,236 (4,647-6,135) | 8,799 (7,010-12,306) |
| Females aged 40-64 | 521 (494-549) | 897 (816-978) |
| Males aged 65 or above | 3,531 (2,326-5,621) | 333 (40-823) |
| Females aged 65 or above | 865 (641-1,121) | 10 (2-16) |

**Table S4: Projected asthma and COPD-related hospitalisation rate**

| **Asthma** | | |
| --- | --- | --- |
|  | **2024** | **2043** |
| Total asthma-specific hospitalisation rate (per 100,000 population) | 72.3 (55.7101.4) | 96.7 (43.8-231.6) |
| **Age-sex subgroups** | | |
| Males aged 0-14 | 192.9 (105.9-351.5) | 321.6 (35.0-1,202.4) |
| Females aged 0-14 | 130.0 (48.8-301.9) | 243.2 (13.8-1,059.3) |
| Males aged 40-64 | 34.5 (29.8-41.3) | 41.5 (22-77.5) |
| Females aged 40-64 | 96.6 (84.7-113.9) | 158.5 (105.6-246.9) |
| Males aged 65 or above | 17.5 (12.7-25.1) | 9.9 (0.3-48.7) |
| Females aged 65 or above | 76.4 (68.1-88.2) | 63.2 (30.6-125.9) |
| **COPD** | | |
|  | **2024** | **2043** |
| Total COPD-specific hospitalisation rate (per 100,000 population) | 60.0 (43.1-109.1) | 59.8 (37.9-90.5) |
| **Age-sex subgroups** | | |
| Males aged 40-64 | 124.5 (108.6-144.1) | 200.5 (127.7-302.5) |
| Females aged 40-64 | 8.4 (7.3-9.8) | 11.8 (6.8-19.2) |
| Males aged 65 or above | 94.4 (66.8-138.1) | 8.7 (10.9-64.6) |
| Females aged 65 or above | 27.0 (4.9-72.9) | 1.6 (0.0-40.1) |

**Figure S3: Projected per-episode costs of multimorbidity in asthma patients**

1. **Hospitalisation costs**

**
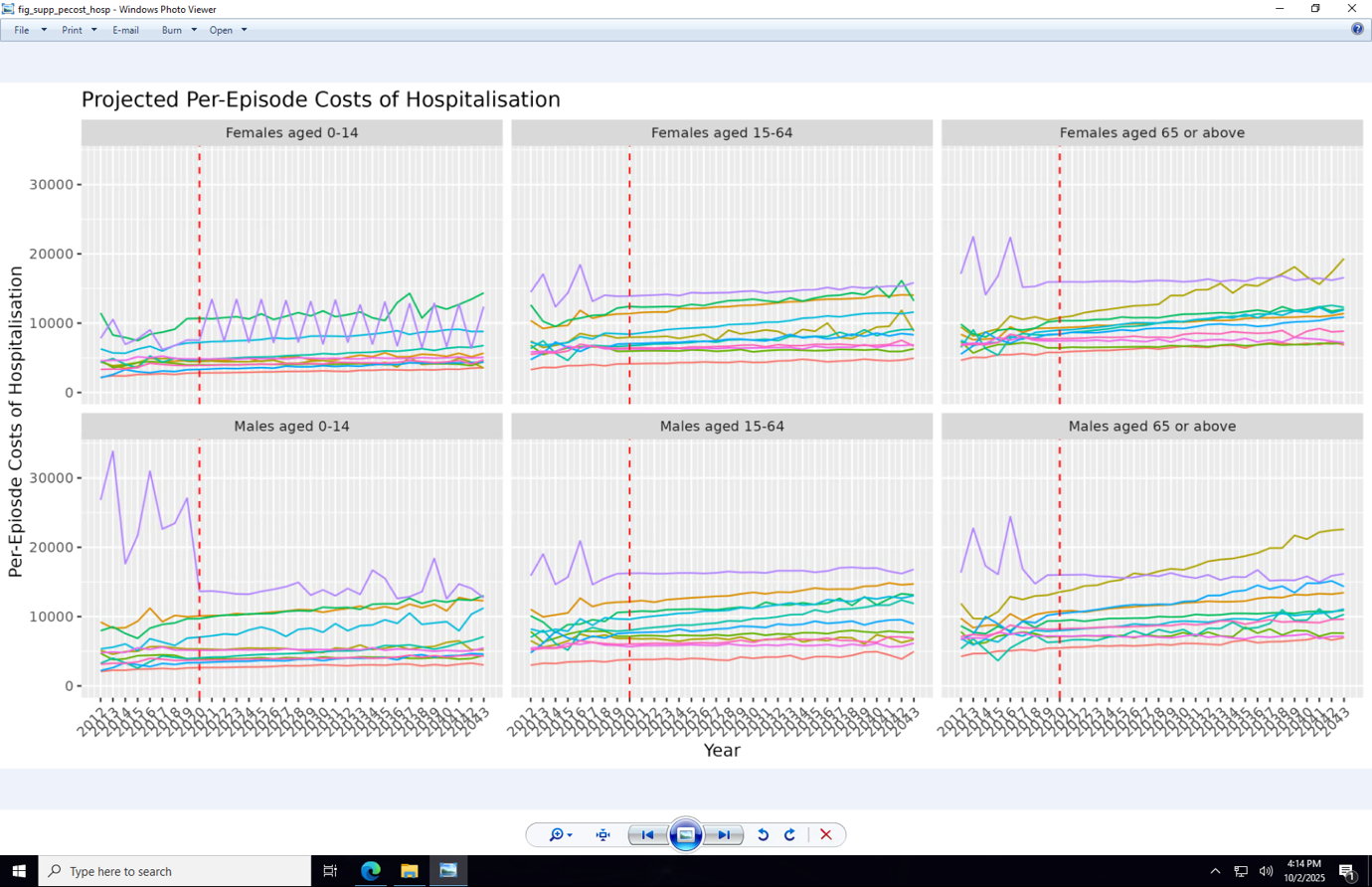
**

1. **ED costs**

**
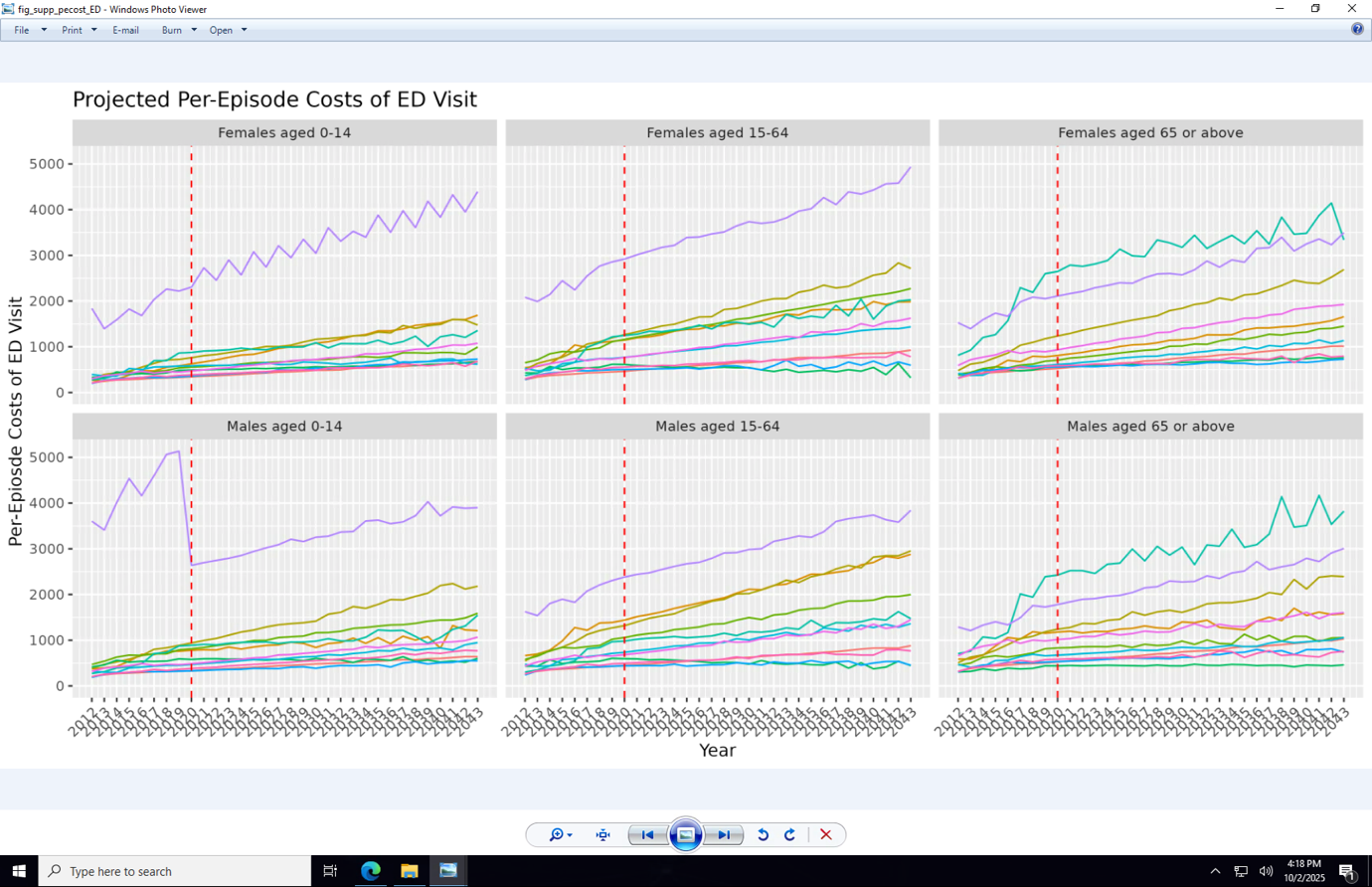
**

1. **Outpatient costs**

**
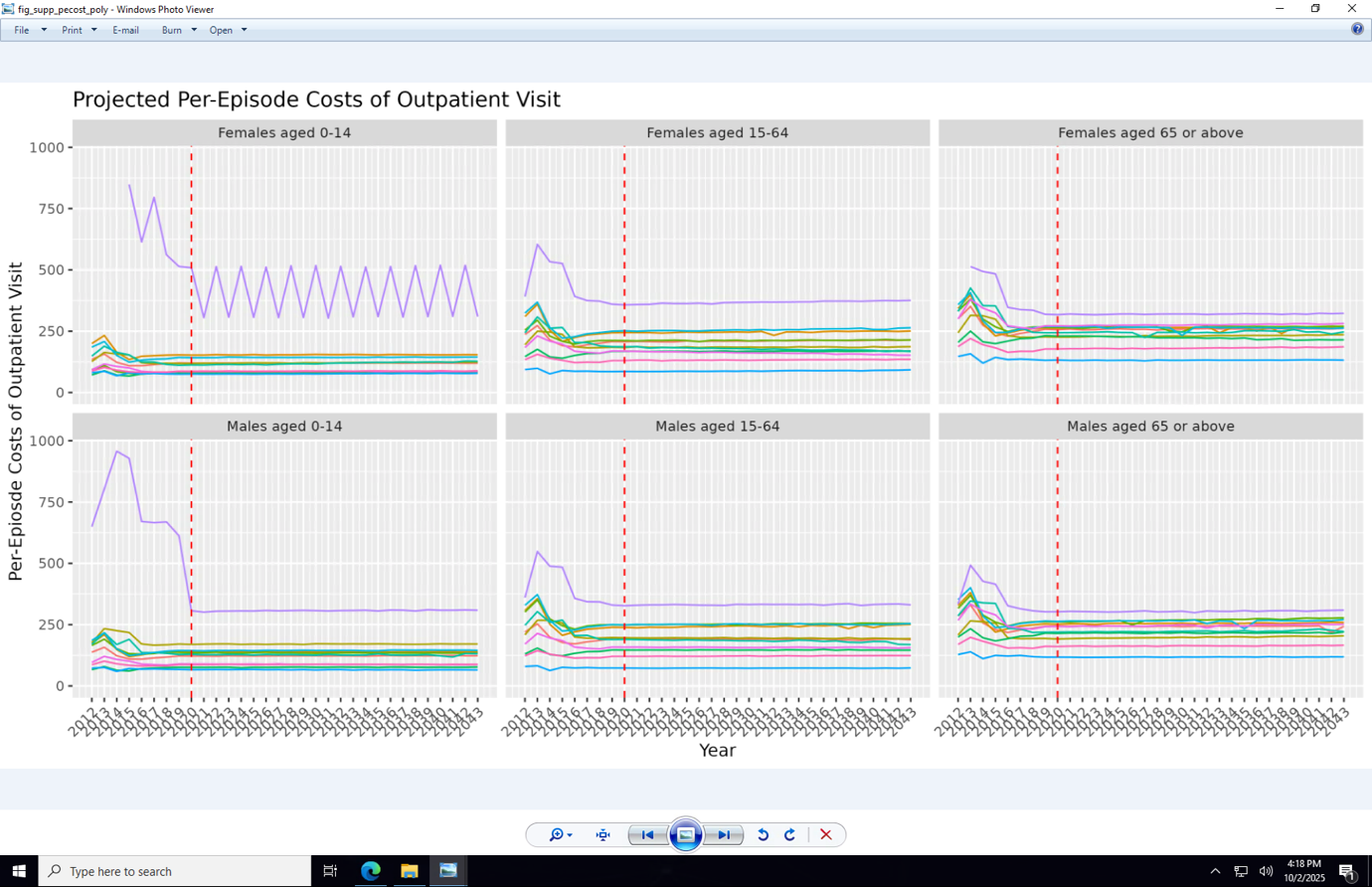
**

**
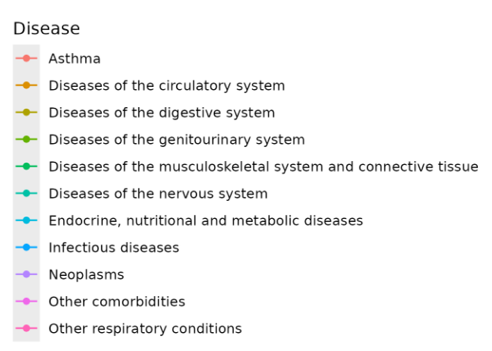
**

**Figure S4: Projected per-episode costs of multimorbidity in COPD patients**

1. **Hospitalisation costs**


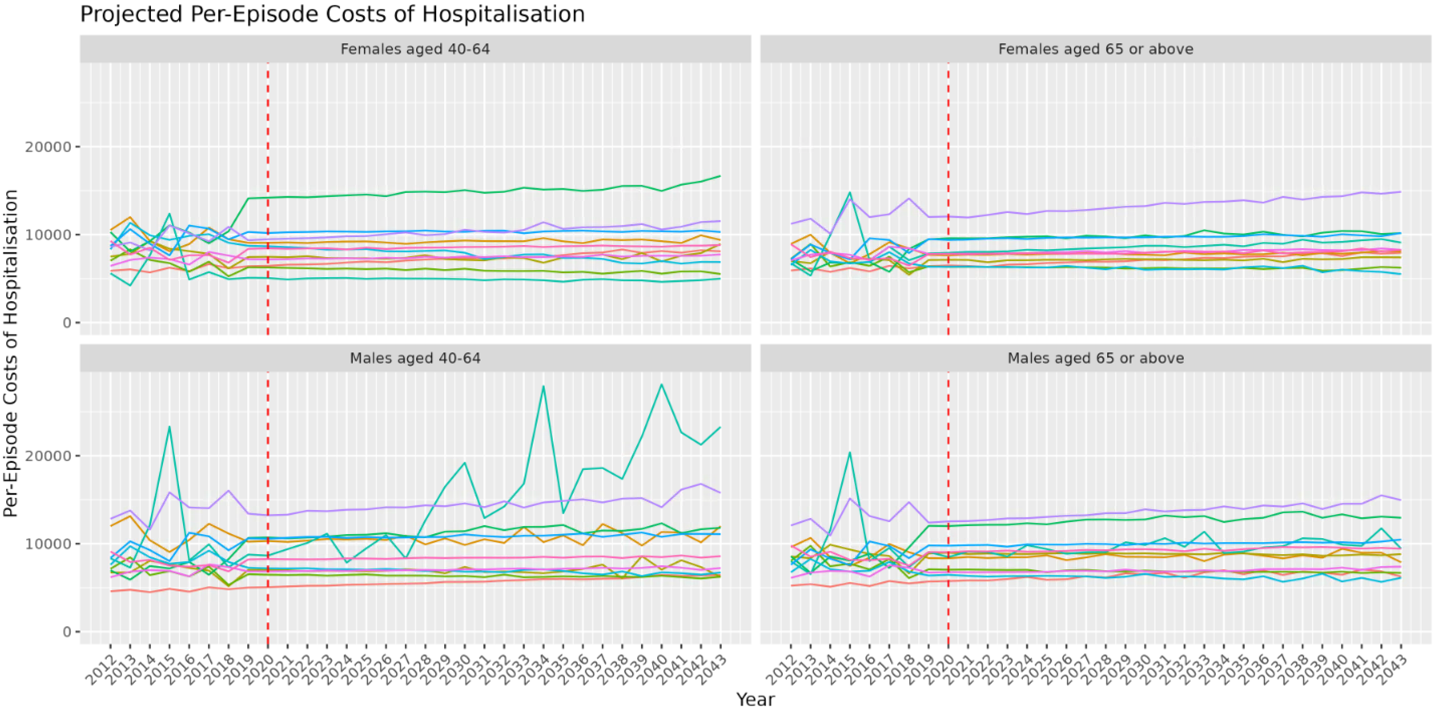


1. **ED costs**


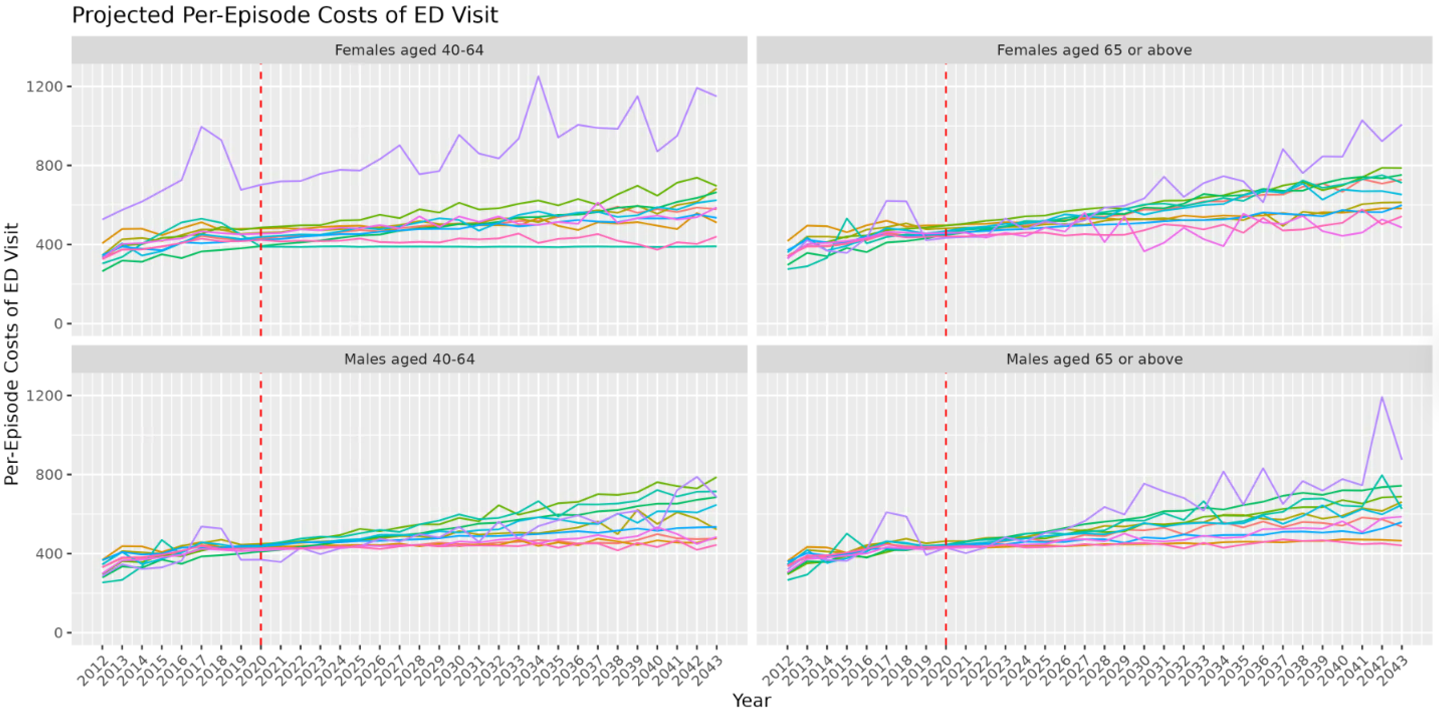


1. **Outpatient costs**


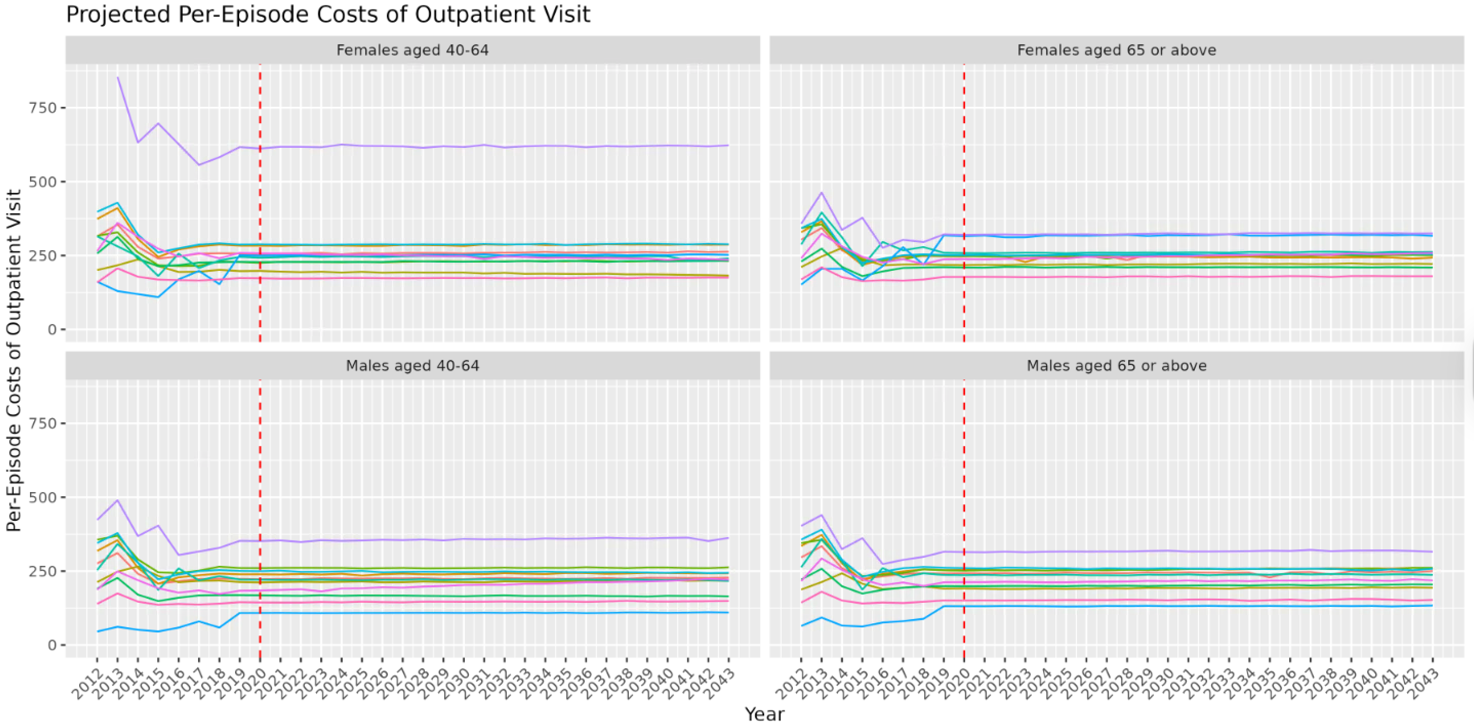


**
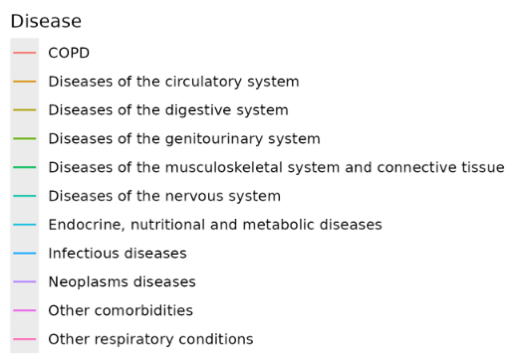
Figure S5: Projected healthcare utilisation rates of multimorbidity in asthma patients**

1. **Hospitalisation rates**

1. **ED visit rates**

**
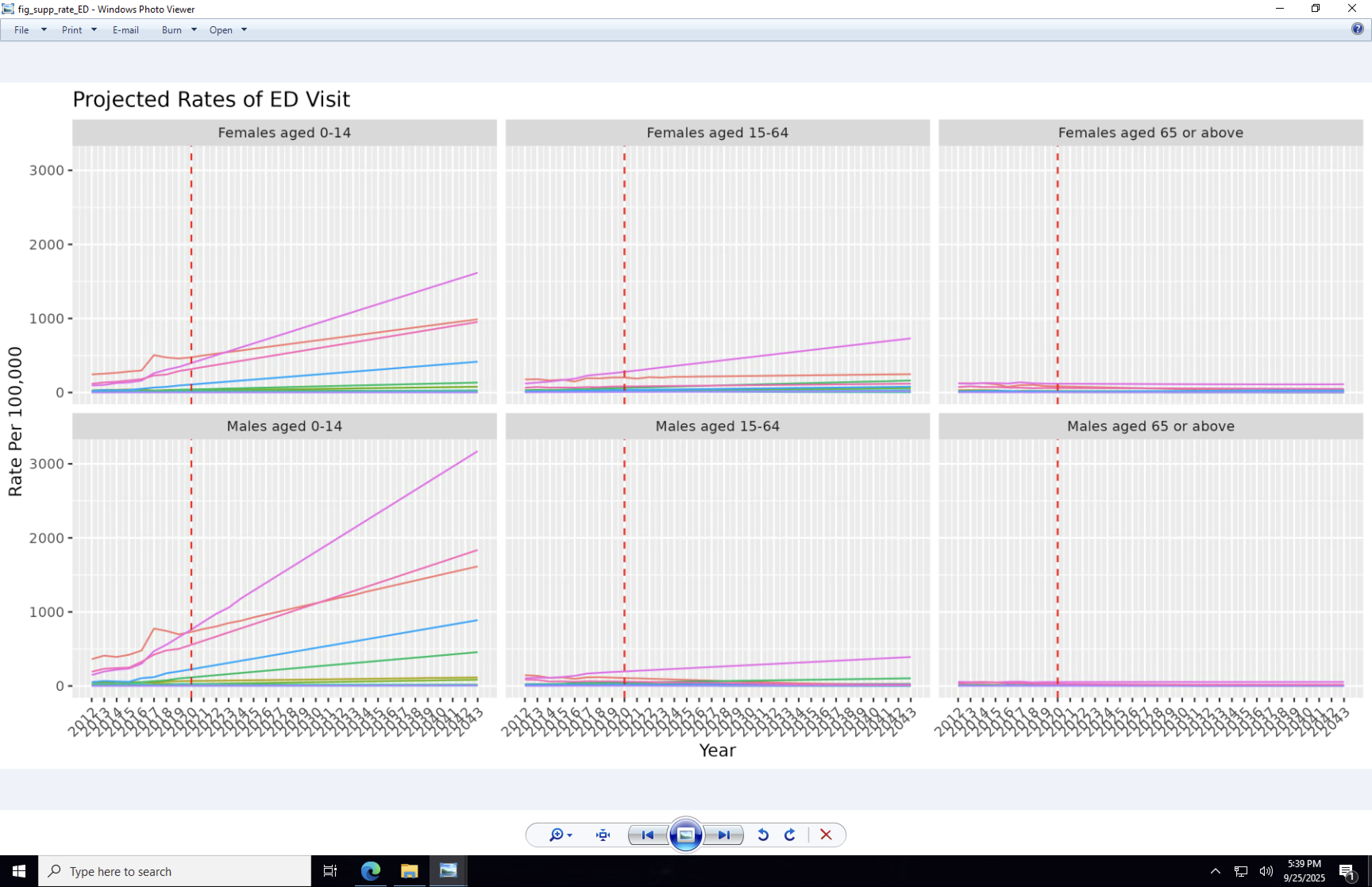
**

1. **Outpatient visit rates**

**
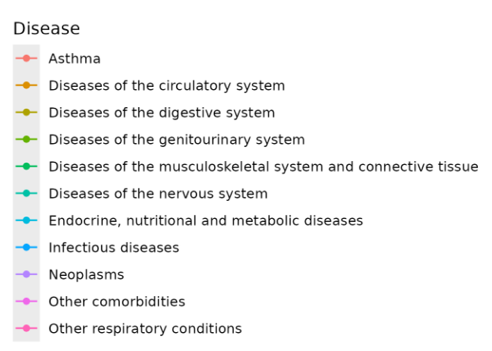
**

**Figure S6: Projected healthcare utilisation rates of multimorbidity in COPD patients**

1. **Hospitalisation rates**

**
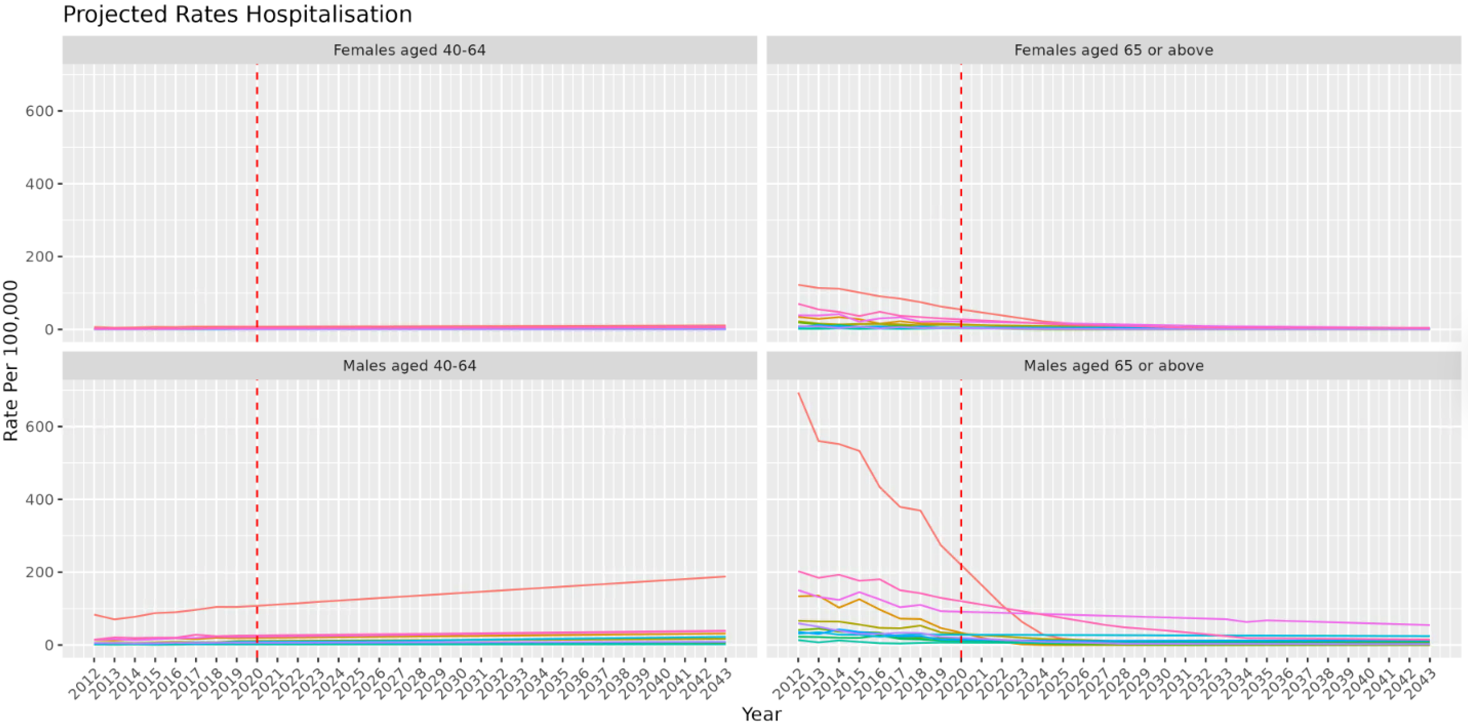
**

1. **ED visit rates**

**
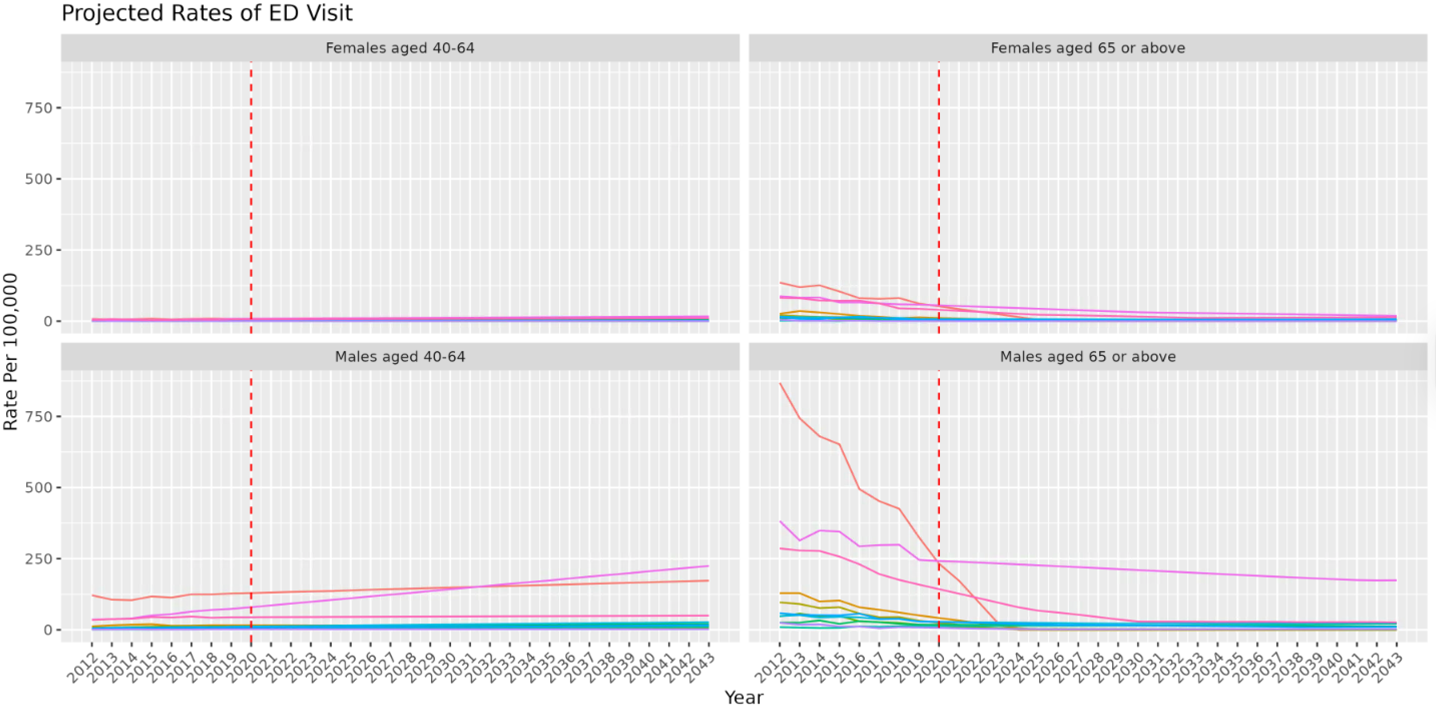
**

1. **Outpatient visit rates**


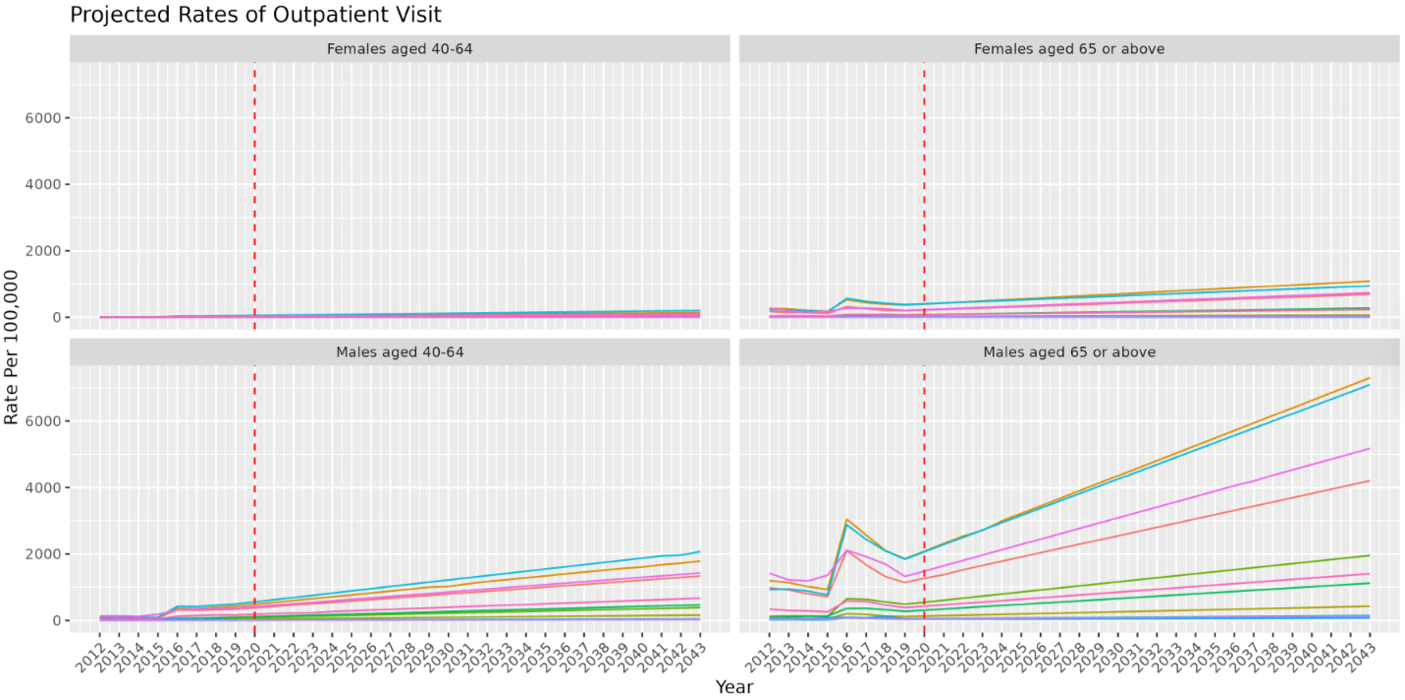


**
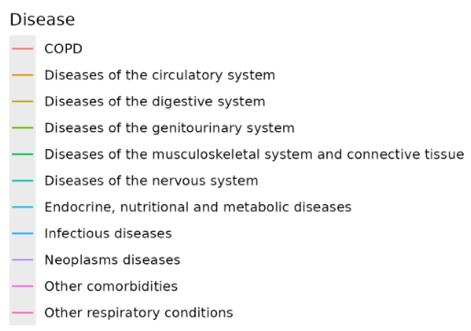
**
